# The socio-cultural significance of mineral licks to the Maijuna of the Peruvian Amazon: implications for the sustainable management of hunting

**DOI:** 10.1186/s13002-020-00412-1

**Published:** 2020-10-07

**Authors:** Michael P. Gilmore, Brian M. Griffiths, Mark Bowler

**Affiliations:** 1 School of Integrative Studies, George Mason University, 4400 University Drive, Fairfax, VA 22030 USA; 2 Environmental Science and Policy, George Mason University, 4400 University Drive, Fairfax, VA 22030 USA; 3 School of Engineering, Arts, Science and Technology Science, University of Suffolk, Waterfront Building, Neptune Quay, Ipswich, IP4 1QJ UK; 4 Institute for Conservation Research, San Diego Zoo Global, Escondido, CA 92027-9614 USA; 5 Suffolk Sustainability Institute, Waterfront Building, Neptune Quay, Ipswich, IP4 1QJ UK

**Keywords:** Mammals, Biodiversity, Wildlife, Traditional ecological knowledge, Ethnoecology, Conservation

## Abstract

**Background:**

The overhunting of wild species is a major threat to biodiversity in the Amazon; yet, managed, sustainable hunting is widely considered part of the solution to conserving wildlife populations. Hunting is both a culturally important activity for Indigenous people and provides an important food source. Mineral licks, a focal point of hunting in Amazonia, are naturally occurring areas in the forest where animals come to obtain essential minerals or clays that are thought to neutralize plant-based alkaloids. We sought to better understand the socio-cultural importance of mineral licks to the Maijuna Indigenous group to inform the sustainable management of this habitat and associated wildlife populations.

**Methods:**

Semi-structured interviews, focus groups, and participatory mapping were carried out with hunters to assess the significance of mineral licks and their associated animal resources as well as to determine how the relationship that the Maijuna have with mineral licks has changed over time.

**Results:**

Mineral licks are culturally significant and useful to the Maijuna in a variety of ways. Hunters target these areas year-round both during the day and night, and animals killed are consumed for subsistence and sold to generate income. The spatial use of mineral licks across the landscape is determined on the generational family level, with families maintaining exclusive use of selected mineral licks and excluding access by other hunters. The Maijuna also have traditional beliefs for why animals visit mineral licks, which is linked to the traditional Maijuna story of the creation of the first tapir. The relationship that the Maijuna have with mineral licks has changed considerably over time, which is observed through changes in hunting technologies and methods as well as the loss of traditional knowledge and beliefs.

**Conclusions:**

Traditional and current Maijuna hunting conventions, in which families maintain exclusive use of selected mineral licks, likely reduce the probability of overexploitation of animal populations. Community-based management plans for mineral licks in Maijuna lands and beyond must incorporate and account for the multiple cultural and economic needs of local communities while also striving toward ecological sustainability. Country-wide strategies to conserving forests and using them sustainably should aim to ensure land tenure for rural peoples and encourage management that incorporates traditional sustainable hunting conventions.

## Background

Hunting threatens wild mammal populations in the Amazon basin [1], but is a culturally important activity for Indigenous people and a principle source of food [2]. The largest animal species are typically the most frequently targeted, with the ungulates like lowland tapir (*Tapirus terrestris*), white-lipped peccary (*Tayassu pecari*), and red brocket deer (*Mazama americana*), and the large rodents, paca (*Cuniculus paca*), usually making up the largest proportion of kills [3]. The larger primates are also widely consumed and are favored by some Indigenous communities [4]. Hunters generally target medium- to large-bodied mammals with shotguns by walking trails during the day or searching with flashlights at night [5].

Most large mammals in Amazonia visit mineral licks. Mineral licks are naturally occurring areas in the forest where animals exhibit geophagical behavior to obtain essential minerals lacking in their diet [6] or clays that relieve indigestion caused by plant-based alkaloids [7]. At undisturbed mineral licks, the ungulates, paca, and ateline primates are the most common visitors [8, 9]. The relatively high frequency of visits by these sought-after game species means that mineral licks are prime locations for hunting, and hunting at mineral licks accounts for up to 30% of all harvested biomass at some sites [10, 11]. Tapir and white-lipped peccary come from afar to use mineral licks, which means that these vulnerable species can be extirpated from a large area simply by concentrated hunting at these focal points [12].

The Maijuna-Kichwa Regional Conservation Area (MKRCA) is one of the Peruvian Amazon’s newest protected areas, comprising 391,039.82 ha of Maijuna Indigenous territory. The Maijuna are one of the smallest and most vulnerable Indigenous groups in Peru [13]. Their ancestral forests sustain and nourish the Maijuna culture, making it critically important to effectively manage and protect the area. In addition to its cultural importance, the MKRCA is one of the most biologically rich places on Earth and an extremely valuable carbon sink [14].

The MKRCA is a powerful example of community-based conservation. The Maijuna originally conceived the idea for conserving this area, and they pushed relentlessly for its creation. After centuries of being marginalized and disempowered by outsiders, the Maijuna established an Indigenous federation in 2004 focused on conserving their lands and culture [13]. Against steep odds, they successfully fought to end exploitive logging and poaching on their lands in 2009 and, with the help of allies, successfully persuaded the Peruvian Government to create the MKRCA in 2015. The Maijuna are committed to effectively manage the MKRCA and its resources. Of particular interest to the Maijuna are medium- to large-bodied mammals, such as tapir and white-lipped peccary, that were severely overhunted by loggers and poachers until 2009.

Understanding the socio-cultural importance of mineral licks to the Maijuna, and the way they are currently and traditionally used are the first steps in understanding how wildlife populations are affected by hunting around mineral licks and whether mineral lick management might be a useful strategy in conserving mammal populations and safeguarding food security in rural Amazonia. Such understanding is also required to ensure that any management plans that are ultimately developed for the MKRCA incorporate and account for the multiple cultural and economic needs of the Maijuna while also striving toward ecological stability. Here we assess the significance of mineral licks and their associated animal resources to the Maijuna and document changing relationships between the Maijuna and mineral licks over time.

## Methods

### Study area

**Fig. 1:**
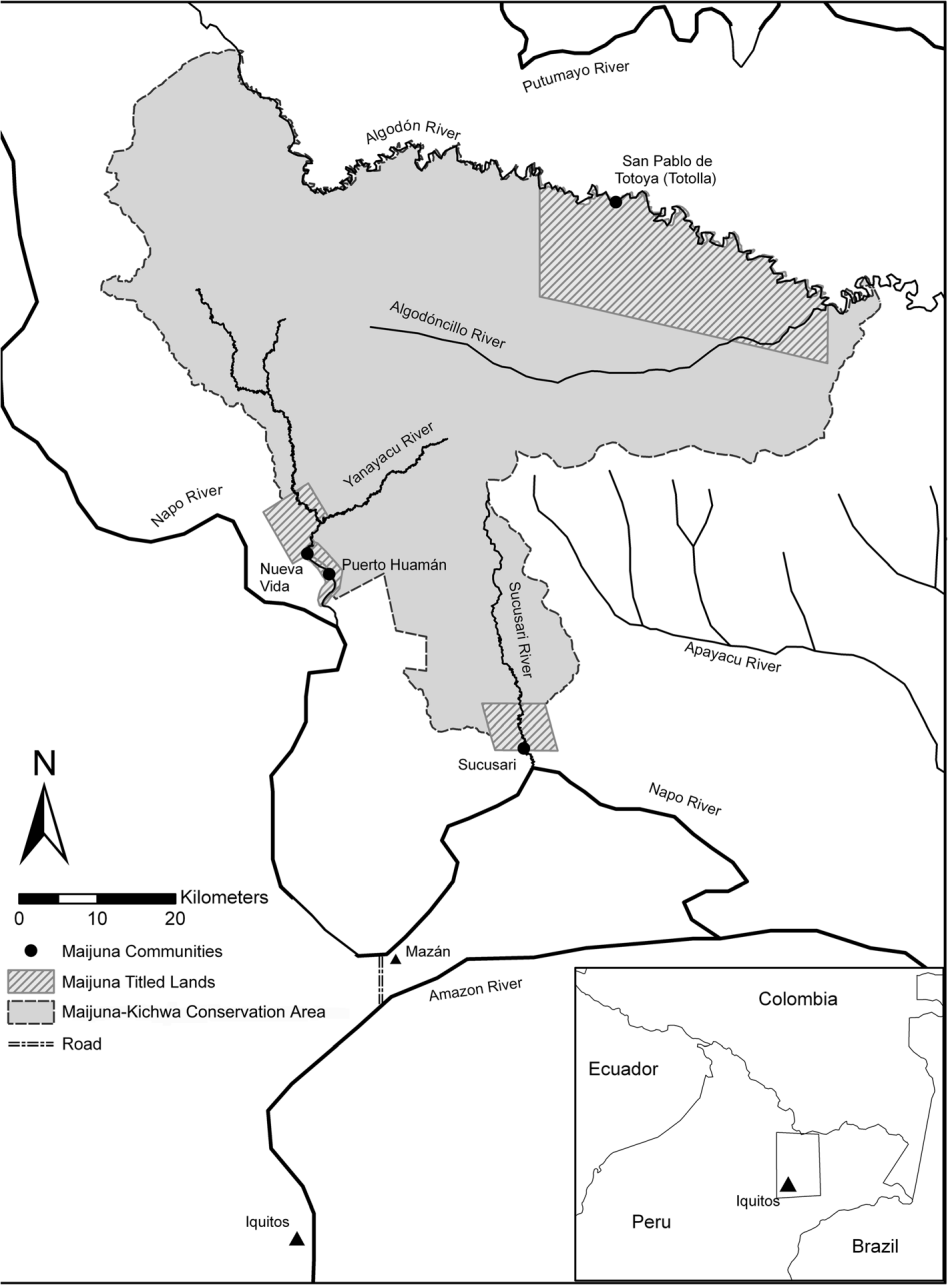
Map of the study area, including all four Maijuna communities and the Maijuna-Kichwa Regional Conservation Area (MKRCA). All field research was conducted in the Maijuna community of Sucusari

The Maijuna, also known as the Orejón, are a western Tucanoan people of the northeastern Peruvian Amazon [13]. Today, there are fewer than 500 individuals living in four Maijuna communities: Puerto Huamán and Nueva Vida along the Yanayacu River, Sucusari along the Sucusari River, and San Pablo de Totoya (Totolla) along the Algodón River (Fig. 1). These three river basins, part of the ancestral territory of the Maijuna, are now within the MKRCA with the headwaters of the rivers in the core of the reserve. No other communities are located within these river basins, which makes supporting and empowering the Maijuna to effectively manage and conserve the MKRCA invaluable to the protection of this immense and extremely biologically and culturally rich area [14].

Data for this study was collected from 1999 to 2019 in the Maijuna community of Sucusari (Fig. 1). This area is approximately 126 km by river from Iquitos, the capital of the Region of Loreto. However, this trip can be shortened to 70 km by crossing the thin isthmus between the Napo and Amazon Rivers by road at Mazán, a small town. The Sucusari community has legal title to 4771 ha that adjoins the recently established MKRCA [14]. The community has 166 residents living in 32 monofamilial or plurifamilial households [15]. Within the residents of Sucusari, 59% are Maijuna, 35% are mestizos^1^, and the remaining 6% are Kichwa. The main subsistence activities of community members include hunting, fishing, swidden-fallow agriculture, and gathering of various forest products. To support basic household needs, residents sell game meat, domesticated animals, agricultural products, and a variety of non-timber forest products [14]. Game meat and other products are sold within Sucusari and in communities nearby along the Napo River as well as in Mazán and the city of Iquitos.

Sucusari is located in an area dominated by upland tropical rainforest, permeated by rivers that are largely bordered by seasonally inundated floodplain forest [20]. Like much of the central Amazon basin, the terrain is relatively flat, with an elevation varying from 80 to 200 m above sea level [21]. This region of the Peruvian Amazon is tropical, humid, and warm with a mean annual precipitation of approximately 3100 mm per year and mean annual temperature of 26 °C [22].

### Data collection and analysis

Prior informed consent (PIC) was obtained from the Sucusari community as well as from individual research participants before beginning the interviews for this study. From 1999 to 2019, semi-structured interviews [23] were conducted with 24 hunters (mean age = 45.04; range = 22–70) to gather information on the significance of mineral licks and their associated animal resources as well as to document changing relationships between hunters and mineral licks over time. The 24 hunters interviewed represent 45.28% of all hunters in the Sucusari community during this 20-year time span. Only men hunt in the Maijuna communities, and a hunter was defined as someone who has hunted at least once in their lifetime; however, all hunters interviewed for this study have hunted extensively throughout their lives. Individual interviews were conducted in Spanish or the Maijuna language, the latter with the help of a community leader that is fluent in both.

Additionally, in July 2017, a focus group was conducted with 16 hunters (55.17% of hunters in the Sucusari community during this time period; mean age = 42; range = 22–68) to identify the locations of mineral licks in the Sucusari River basin using participatory mapping techniques [24, 25]. Mapped mineral licks were then visited, and their locations marked using a hand-held GPS unit to create an accurate base map of the mineral licks of the Sucusari River basin. During an 11-month period from August 2018 to June 2019, this base map was then used to interview 19 active hunters (91% of active hunters in the Sucusari community during this time period; mean age = 41; range = 22–68) on a weekly basis about their hunting routes and the mineral licks that they visited. This map was continually updated during this time period as new mineral licks, trails, streams, and hunting camps were identified and mapped with a GPS. To identify key themes, perceptions, and issues in the data collected, qualitative data were coded, organized, and analyzed using a grounded theory framework based on the methods described by Corbin and Strauss [26].

## Results and discussion

### Classification and use of mineral licks

Mineral licks are called ***tùàrà***^2^ (mineral lick) or ***ónóbɨ*** (place of *masato*) in the Maijuna language and *collpa* in the local Spanish dialect. Mineral licks are one of dozens of different forest habitat types defined and classified by the Maijuna. They classify forest habitats based on geomorphology, physiognomy, disturbance, indicator plant species, and indicator animal species [20]. According to the Maijuna, mineral licks are found at the base of hillsides in both floodplain and upland forest, have a “face” where animals come to consume soil or drink water, and are poorly drained and muddy areas; all of which follows the Western scientific understanding of these areas as well [9, 28]. In short, mineral licks are both culturally defined and classified by the Maijuna as is the case in other Indigenous and non-Indigenous communities in the Peruvian Amazon [29, 30].

Mineral licks are culturally and economically important to the Maijuna because a number of game animals visit these sites and hunters target these areas year-round for hunting. These mammal and bird species are either diurnal or nocturnal; therefore, Maijuna hunters target mineral licks both day and night revealing an important temporal relationship with these areas (Table 1). Hunted species at mineral licks include some of the most important game species in Maijuna lands, including paca (*C. paca*), collared peccary (*T. tajacu*), white-lipped peccary (*T. pecari*), red brocket deer (*M. americana*), and tapir (*T. terrestris*), all of which are well documented in the literature for visiting mineral licks [8, 9, 28]. The meat of these species is not only consumed for subsistence, but it is also sold to generate income. Parts of these animals are also used in the making of tourist crafts, traditional medicines, and musical instruments, among other things.

**Table 1:** Game animals encountered and killed by the Maijuna at mineral licks [20, 24, 25]

Species	Maijuna name	Local name	English name	Time encountered (day/night)	Use
**Birds**					
*Pipile cumanensis*	***újé***	*pava*	Blue-throated piping-guan	Day	Eat, sell (meat), used to make fans for fires (feathers), adornment (make “paint” from legs)
**Mammals**					
*Cuniculus paca*	***sèmè, gójébèkò***	*majás*	Paca	Night	Eat, sell (meat), tourist crafts (teeth)
*Alouatta seniculus*	***jáíkɨ***	*coto mono*	Red howler monkey	Day	Eat, sell (meat), tourist crafts (bony pouch or hyoid bone from throat)
*Coendou prehensilis*	***tòtò***	*cashacushillo*	Brazilian porcupine	Night	Eat, tourist crafts (spines)
*Dasyprocta fuliginosa*	***máítàkò, kòròmé***	*añuje*	Black agouti	Day	Eat, sell (meat), tourist crafts (teeth)
*Mazama americana*	***bósá***	*venado colorado*	Red brocket deer	Night, rarely during day	Eat, sell (meat), medicinal (antlers), adornment of houses (antlers), used to make drums (hide)
*Tapirus terrestris*	***békɨ, jáíkò***	*sachavaca*	Lowland tapir	Night	Eat, sell (meat), medicinal (hooves), tourist crafts (hooves)
*Tayassu pecari*	***bɨrɨ, bàì***	*huangana*	White-lipped peccary	Day	Eat, sell (meat and hide), tourist crafts (teeth), used to make drums (hide)
*Pecari tajacu*	***káókwà***	*sajino*	Collared peccary	Day	Eat, sell (meat and hide), tourist crafts (teeth), used to make drums (hide)

Hunting at mineral licks takes several forms. While walking trails on hunting trips during the day and night, hunters will quickly stop by mineral licks along their routes to see if they can opportunistically catch game species in the lick and to also check for signs of animal activity (i.e., animal tracks). Additionally, hunters will wait alone for several hours at a time at licks with signs of animal activity during both the day and night. They lie in wait at strategic spots above licks with good views and clear shots of the area. At these strategic spots, they will either sit on the ground, make a hunting platform, or string up a hammock between two trees. At night, they lie in wait with their flashlight off listening for animal activity and, according to hunters, they can tell the species entering the lick by the amount and type of noise that it makes as it walks through the mud and standing water to the lick “face.” If it is a desirable species, the hunter will then attempt to ambush the animal by turning on their flashlight when it has reached a desirable location in the lick with a clear shot.

**Fig. 2:**
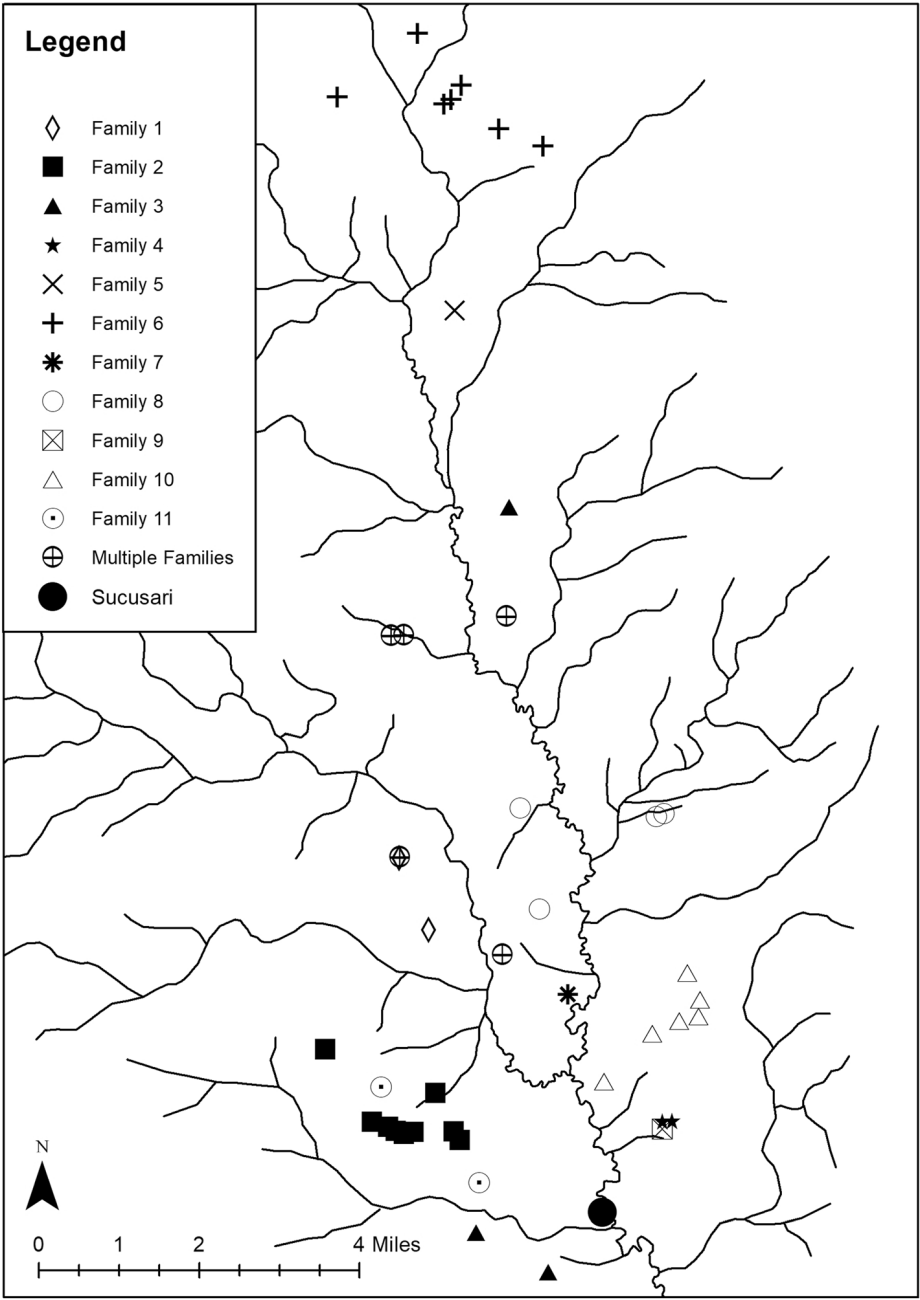
Forty-three mineral licks visited by hunters in the Sucusari River basin during an 11-month period from August 2018 to June 2019. Mineral licks are divided by family use

Eighty-four mineral licks were identified and had their locations fixed by GPS in the Sucusari River basin during this study. Of these, 43 were regularly visited by hunters during the 11-month period from August 2018 to June 2019. However, during this time period, all hunters did not regularly or equally visit each of these 43 licks. Instead, clear spatial use of 38 of these mineral licks is observed when analyzing the data by family, with each family having distinct mineral licks that they regularly hunt at that others do not enter (Fig. 2). A family, for the sake of this analysis, was defined as a group of individuals united by ties of blood, adoption, or marriage that live in the same household, with some households having multiple hunters that hunt at the same licks. For example, one family during this time period was composed of four individuals, a mother and father with two grown sons (ages 28 and 33) that hunt at the same mineral licks (family 6, Fig. 2). However, the older brother (age 45) of these two hunters lives in a different household with his own wife and children and hunted at totally different mineral licks (family 11, Fig. 2). Although 100% of the hunters interviewed during this 11-month period said that they can hunt at whatever mineral lick they want in the Sucusari River basin, including in another family’s licks, clear spatial segregation is in fact carried out and observable. Knowledge of mineral licks is passed down through generations, although a hunter marrying into another family will often be taken to the family’s licks. This spatial segregation of licks demonstrates the power of traditional resource use rights and strategies. The mineral licks that individual families do hunt at tend to be accessible by trail from either their primary residence or hunting camp(s), with hunting camps being located in more remote parts of the river basin. It is worth noting that only five mineral licks out of the 43 frequented during this time period were regularly visited by multiple families (Fig. 2). These mineral licks tend to be very close to well-known and regularly used hunting camps and are easier to access.

Due to their use and significance to the Maijuna, mineral licks appear to be a textbook example of what Posey ([31]: 117) referred to as “resource islands.” According to Posey [31], “resource islands” are “…areas in the primary forest where specific concentrations of useful plants or animals are found.” Through his work with the Kayapó of the Brazilian Amazon, Posey [31] provides several general examples of “resource islands,” including sources of palm hearts, palmito and palm nut sources, areas with cane for arrows, hunting areas, and fish concentrations, among others. Conceiving of mineral licks as “resource islands” distributed across the landscape ultimately helps to highlight the importance of properly managing and conserving these ecologically, culturally, and economically significant areas in Maijuna lands.

### Mapping and naming of mineral licks

**Fig. 3:**
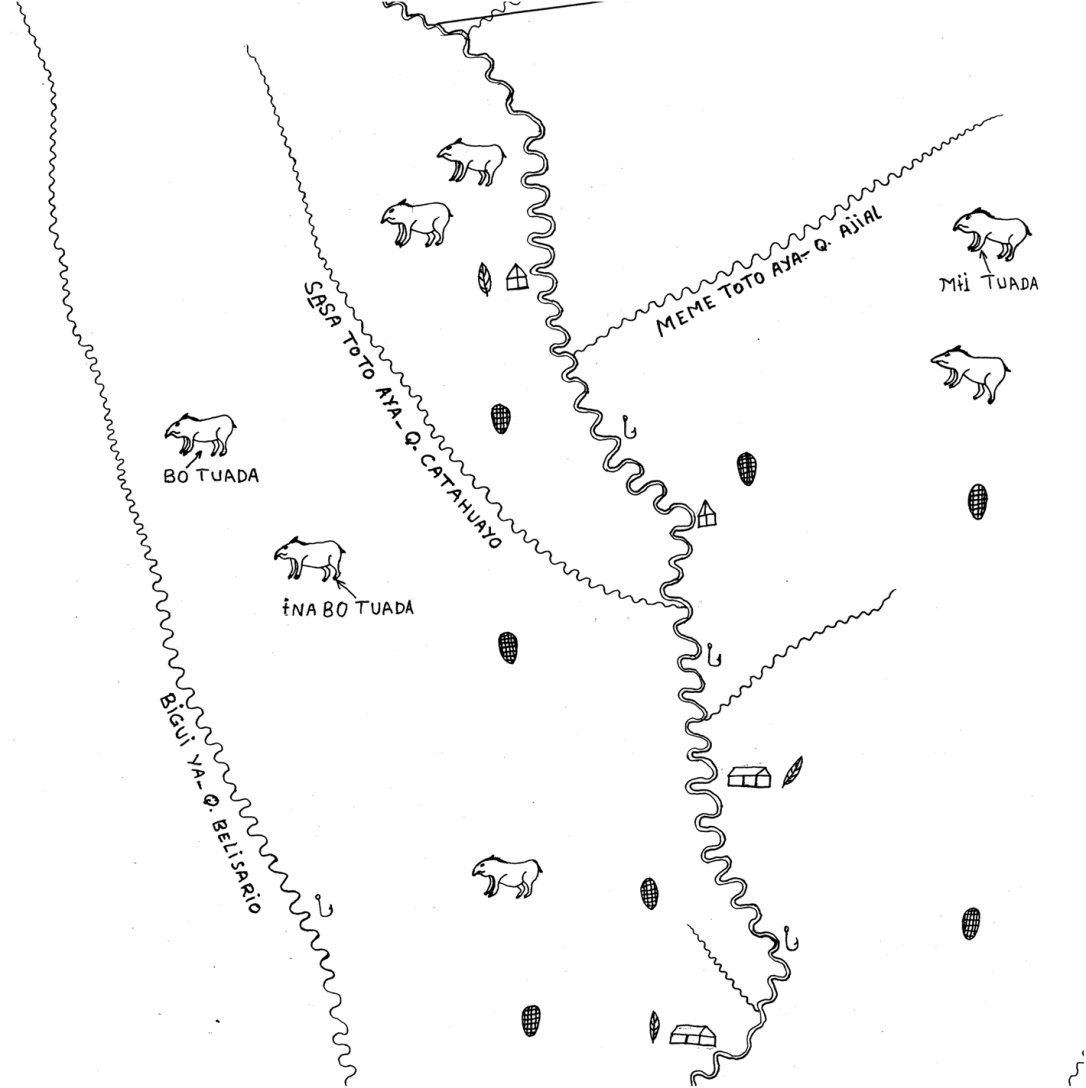
Portion of a participatory map drawn by Sucusari community members in 2004. Participants chose the tapir symbol to indicate the locations of mineral licks throughout the river basin. The traditional Maijuna names of three mineral licks are indicated

A separate participatory mapping project carried out with the Maijuna between 2004 and 2009 (see [24, 25, 32–34]) further highlights the cultural significance of mineral licks. During mapping sessions in all four communities, mineral licks were consistently one of the first bioculturally significant sites that participants mapped highlighting the cultural salience and importance of these areas. In the Sucusari community, after discussing and comparing different ideas [32], mapping participants selected the symbol of a tapir to represent the locations of mineral licks throughout the river basin on their hand-drawn map (Fig. 3). According to participants, the tapir was chosen as the symbol for mineral licks given that it is one of the most culturally important game species that visits mineral licks as well as its key role in a traditional story detailing the creation of the first lick (see below).

Out of the 84 mineral licks that were ultimately identified in the Sucusari River basin during this study, 21 have proper names in the Maijuna language. The Maijuna name mineral licks after plants found in or around the area, individuals who hunted there, birds or bats that frequent the lick, general vegetative characteristics, soil type, biting ants present, and the size of the area. The extensive naming of mineral licks further highlights their significance to the Maijuna [20, 24, 25, 35].

### Traditional beliefs about mineral licks

The Maijuna have a very different explanation for why animals visit mineral licks [36]. According to the traditional Maijuna story of the creation of the first tapir, a man named ***Békɨtù*** was seeking revenge on his son-in-law. He tried to trick him three times, yet his son-in-law outsmarted him each time. After the third time, the son-in-law used his powers as a creator to turn ***Békɨtù*** into a tapir. During this slow transformation, ***Békɨtù*** began to take on more and more qualities of tapirs; he increased in size, made similar noises, began to eat *Mauritia flexuosa* fruits whole, and became skittish around people. Toward the end of this transformation, ***Békɨtù*** asked his daughter, who was unaware of what was happening, to make *masato* (a traditional alcoholic beverage made from *Manihot esculenta*) and bring it to him in the forest. When his daughter returned, he grew increasingly skittish, and eventually refused to come close when she approached, despite her repeated assurances. The exasperated daughter threw her clay pot of *masato* on the forest floor, where it shattered. ***Békɨtù*** later came and licked up the *masato*, using his hooves to further mix up the brew and ultimately soften the moist earth, the very first tapir forming the very first mineral lick. In the Maijuna language, the word for *masato* is ***ónó*** and the word for place is ***bɨ***. A mineral lick, ***ónóbɨ***, literally means “place of *masato*,” which is why animals are attracted. The Maijuna believe that the different animal species that visit mineral licks are there specifically to drink *masato*.

### Changing relationships through time

First contacted by Jesuit missionaries in 1682, the Maijuna’s population declined dramatically due to epidemics, fighting, and then enslavement during the rubber boom. Over the years, Maijuna traditional beliefs, language, and ways of life have been undermined by missionary teachings, *patrones*^3^, regional society, ill-conceived government policies, and a formal education system that does not value traditional knowledge [13, 37, 38]. This has led to a disconnect with their traditional cultural practices and beliefs, causing a rapid loss of traditional knowledge [13–15, 20, 35], commonly recognized as acculturation [39].

Due to these realities, the relationship that the Maijuna have with mineral licks has changed considerably over time. For example, they traditionally hunted game animals at mineral licks with spears only during the day. Over the past 100 years, the introduction of shotguns in the Maijuna communities has drastically increased hunting efficiency over traditional methods [40]. The acquisition of incandescent battery-powered flashlights has also facilitated night hunting, allowing the harvesting of nocturnal species at mineral licks. More recently in approximately 2011, the Maijuna began using new, brighter, and more efficient light-emitting diode (LED) flashlights enabling hunters to find and kill prey more easily by increasing the freezing response of many species in spotlights. Maijuna hunters now hunt longer and more frequently at night, in mineral licks and beyond, increasing kill rates of nocturnal species and likely overall harvests [5]. All of these changes in hunting technologies and methods, along with increasing integration into the market economy and subsequent hunting for income generation, have significant conservation and management implications for the hunting of game animals at mineral licks in Maijuna lands.

The Maijuna’s changing relationship with mineral licks is also evident in both the naming of and traditional beliefs about licks. Out of the 19 active hunters interviewed for this study in the Maijuna community of Sucusari, only four (21%) know the names of mineral licks in the Maijuna language. The rest use exclusively Spanish names for mineral licks in the Sucusari River basin, which are sometimes direct translations from the name in Maijuna but most of the time are not. This is not only due to the fact that the Maijuna language is highly endangered and in disuse by younger Maijuna generations [13], but also due to the fact that 9 out of the 19 active hunters (47%) are not Maijuna and instead are mestizo or Kichwa.

Additionally, although most of the 10 active hunters that are Maijuna know of or have heard the traditional Maijuna story of the creation of the first tapir, only two (ages 65 and 68) know the story in significant detail and are able to tell it in the Maijuna language. This loss of knowledge and traditional beliefs is significant given that traditional stories symbolize unity and the creation of shared bonds for Indigenous people [41]. Most traditional stories help to provide the ethical and moral foundations on which Indigenous cultures are built, document the creation of ancestral lands, and/or disentangle natural events [42, 43]. These stories reinforce the connection Indigenous people have with nature by highlighting the significance of natural resources for the survival of living beings [44, 45]. The Maijuna traditional story of the creation of the first tapir helps to explain not only the origin of this important game species but also a culturally significant habitat and hunting site that they depend on for sustenance and survival. Additionally, the duality of animals and people in this story may represent the measured use of wildlife through cultural beliefs and traditions, a form of adaptive management of wildlife populations [46, 47].

## Conclusions

Through interviews, focus groups, and participatory mapping, we were able to gain a comprehensive understanding of the importance of mineral licks to the Maijuna. Mineral licks are important hunting areas for both subsistence and income generation, and their spatial use across the landscape is determined on the family level. Additionally, the classification and naming of licks as well as traditional beliefs about them further highlights their socio-cultural significance to the Maijuna. Learning all of this information is an essential first step for the development of community-based management plans for mineral licks in the MKRCA that incorporate and account for the multiple cultural and economic needs of the Maijuna while also striving toward ecological sustainability. Next steps could include large-scale monitoring of wildlife use of licks, examining the impacts of hunting at mineral licks on wildlife, and assessing the potential of working with local communities to protect licks as a strategy to manage hunting and conserve wildlife populations. Such management strategies are likely to be similarly useful across the wider Amazon basin, where mineral licks are also of considerable cultural and economic importance as hunting sites [8, 10].

Traditional and current Maijuna hunting conventions, in which families maintain exclusive use of selected mineral licks, and exclude access by other hunters, likely reduce the probability of overexploitation, especially where hunting is for subsistence rather than commercial purposes. Where Indigenous or local people do not have ownership and control of their hunting grounds, more frequent use of mineral licks by more hunters may occur, increasing the chances of over extraction of wild species. Indigenous land title and the right to restrict access to hunting grounds are key to tackling overhunting in Amazonia.

Although it was found that traditional knowledge and beliefs about mineral licks is in steep decline, the Maijuna still remain deeply connected to their ancestral lands and rely heavily on licks and their associated game species. The role and importance of Indigenous people in conservation is increasing [48–51], which is demonstrated by the key role of the Maijuna in not only the creation of the MKRCA but the ongoing management of this heavily forested and biodiverse area. As community-based management plans for mineral licks and associated game species are developed for the MKRCA, it is critical that these efforts also address the conservation and revitalization of traditional knowledge and beliefs about these areas. This would help to ensure that both the biological and cultural significance of mineral licks to the Maijuna are being targeted. Given their ecological importance, status as “resource islands,” and the traditional beliefs about them, mineral licks in Maijuna lands are complex biocultural systems and need to be managed in a holistic and integrative manner. Management plans that build on and strengthen traditional knowledge and traditional resource use rights and strategies would help to ensure the maintenance of dynamic relationships between mineral licks, their associated biological diversity, and the hunters that use them. Mineral licks are important hunting sites not only in Maijuna lands but also throughout the Amazon basin, and we believe our study provides a much-needed in-depth investigation of how hunters and communities interact with this culturally, biologically, and economically important habitat.

## Data Availability

The datasets used and/or analyzed during the current study are available from the corresponding author on reasonable request.

## Abbreviations

LED: Light-emitting diode
MKRCA: Maijuna-Kichwa Regional Conservation Area
PIC: Prior informed consent

## Footnotes

1 *Mestizos* are people of mixed Amerindian and Iberian descent found throughout the Peruvian Amazon who practice a mixture of traditional agriculture, hunting, fishing, and forest product extraction for their livelihoods ([16–18] as cited in [19]: 421)

2 All Maijuna terms are in boldface italics. Transcription of Maijuna words was accomplished with the help of Sebastián Ríos Ochoa, a literate and bilingual Maijuna individual, using an orthography developed by Michael et al. [27]. The spelling of Maijuna words was then crosschecked using this same source.

3 *Patrones* are colonists and their descendants who exploited Indigenous labor to harvest forest resources.
